# Primary Small Intestinal Adenocarcinoma Presenting as Ovarian Metastasis With Obstructive Symptoms: A Case Report

**DOI:** 10.7759/cureus.106358

**Published:** 2026-04-03

**Authors:** Yusuke Akimoto, Kenjiro Ishii, Osahiko Hagiwara, Kiribayashi Takaharu, Toshiyuki Enomoto, Koji Asai, Takuya Nagata, Manabu Watanabe, Yoshihisa Saida

**Affiliations:** 1 Department of Surgery, Toho University Ohashi Medical Center, Tokyo, JPN; 2 Department of Surgery, Toho University Ohashi Medical Center Ohashi Hospital, Tokyo, JPN

**Keywords:** jejunal cancer, krukenberg tumor, ovarian metastasis, small intestinal adenocarcinoma, ulcerative colitis

## Abstract

Primary small-bowel adenocarcinoma (SBA) is a rare malignancy. As early symptoms are nonspecific, diagnosis is frequently delayed, and patients often present with advanced disease. Ovarian metastasis from SBA - classified as a Krukenberg tumor - is exceptionally uncommon and may obscure the underlying gastrointestinal origin, posing a significant diagnostic challenge.

We report the case of a 39-year-old woman with a history of ulcerative colitis who presented with abdominal distension and was found to have a rapidly enlarging left ovarian mass. Left adnexectomy revealed adenocarcinoma, and further evaluation identified a circumferential ulcerative lesion in the upper jejunum. Immunohistochemical analysis demonstrated CK7 positivity, CK20 positivity, CDX2 positivity, and PAX8 negativity in both ovarian and jejunal specimens, confirming a diagnosis of primary jejunal adenocarcinoma with ovarian metastasis. The patient subsequently underwent laparoscopic partial jejunal resection. Histopathology confirmed stage IV disease (pT3 NX M1). Postoperatively, capecitabine and oxaliplatin (CAPOX) plus bevacizumab chemotherapy was initiated following colorectal cancer protocols.

This case illustrates several diagnostic challenges associated with SBA, particularly when presenting as an ovarian mass. Ovarian metastasis from SBA is exceedingly rare, and the presence of bilateral, non-synchronous disease further complicates diagnosis. Immunohistochemistry played a key role in distinguishing the jejunum as the primary site. Ulcerative colitis may have contributed to carcinogenesis. Despite therapeutic advances, stage IV SBA remains associated with poor prognosis.

This rare case of primary upper jejunal adenocarcinoma presenting as a Krukenberg tumor underscores the importance of thorough diagnostic evaluation, including immunohistochemical analysis. Multidisciplinary management and timely surgical intervention remain essential for both diagnosis and symptom control in advanced SBA.

## Introduction

Primary small intestinal malignancies are rare, accounting for approximately 0.1%-1% of all gastrointestinal tract cancers [1]. Histologically, these tumors include adenocarcinoma, lymphoma, neuroendocrine tumors, and sarcoma, among which adenocarcinoma represents the most common epithelial malignancy. Several protective factors unique to the small intestine, including rapid transit time, dilution of carcinogens by fluid luminal contents, and abundant lymphoid tissue, are thought to contribute to this low incidence. Recent large-scale analyses from Japan have further clarified the epidemiologic characteristics of small-bowel malignancies. Yamashita et al., using the Japanese Society for Cancer of the Colon and Rectum (JSCCR) database, reported that the duodenum is the most common site (52.2%), followed by the jejunum (31.7%) and ileum [2].

Small-bowel adenocarcinoma (SBA) often presents with nonspecific symptoms such as abdominal pain, anemia, nausea, vomiting, and weight loss, frequently resulting in delayed diagnosis and advanced-stage disease at presentation. Physical examination findings may include abdominal distension, tenderness, or a palpable mass in advanced cases. Contrast-enhanced computed tomography (CT) is the primary imaging modality for detecting bowel wall thickening, obstruction, and metastatic lesions. Additional diagnostic modalities, including capsule endoscopy and balloon-assisted enteroscopy, are useful for direct visualization and histologic confirmation of small-bowel lesions. In addition, previous clinicopathological studies have reported that metastatic tumors involving the small bowel may present with similar imaging findings, highlighting the importance of careful evaluation to distinguish primary from metastatic lesions [3]. Surgical resection with regional lymphadenectomy remains the cornerstone of treatment for localized disease, whereas systemic chemotherapy is recommended for advanced or metastatic cases.

The most common metastatic sites of SBA include regional lymph nodes, the peritoneum, and the liver [2,4]. Ovarian metastasis from gastrointestinal malignancies, known as Krukenberg tumors, most frequently originates from gastric or colorectal cancer but may also arise from the small intestine [5-7]. Previous studies have reported more than 70 cases of ovarian metastases from SBA, indicating that although uncommon, this metastatic pattern is well recognized [7,8]. Patients with Krukenberg tumors often present with gynecologic manifestations, such as pelvic masses, abdominal distension, or ascites, which may initially lead to suspicion of primary ovarian malignancy rather than a gastrointestinal origin.

We herein report a case of primary upper jejunal adenocarcinoma identified following surgical resection of an ovarian mass consistent with a Krukenberg tumor. This case highlights the diagnostic challenges associated with atypical metastatic presentation. It underscores the importance of comprehensive imaging, immunohistochemical evaluation, and appropriate surgical management in establishing an accurate diagnosis and treatment strategy.

## Case presentation

A 39-year-old woman with a history of ulcerative colitis, asthma, and cervical cancer treated by conization three years earlier had been followed regularly every six months. There was no family history of malignancy. Serum tumor markers at presentation were elevated (CEA: 10.8 ng/mL; CA19-9: 52.9 U/mL).

**Figure 1:**
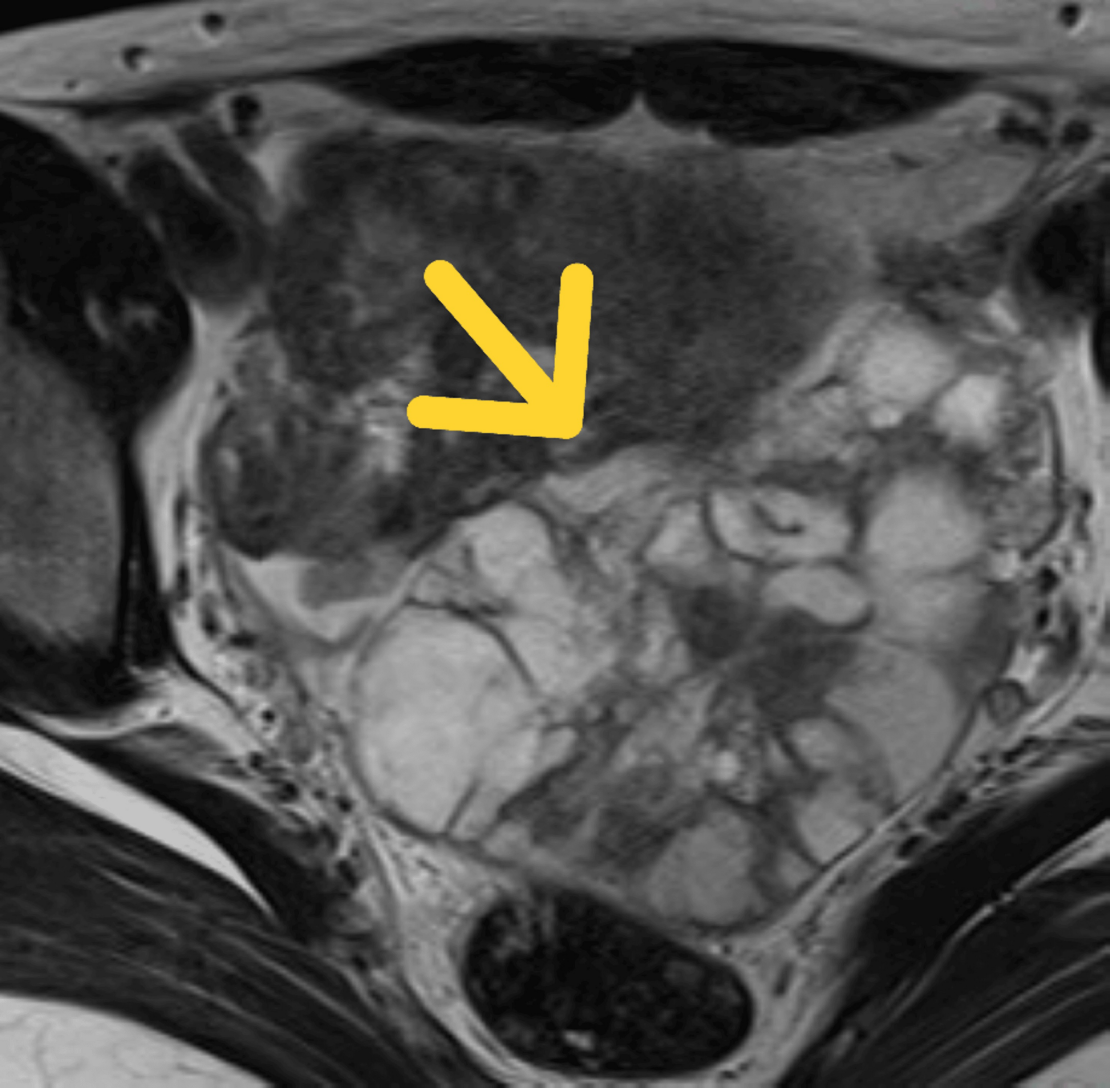
Axial T2-weighted MRI showing the ovarian mass Axial T2-weighted MRI demonstrating a 106 × 68 mm multilocular cystic ovarian mass with internal septations and heterogeneous high signal intensity, raising suspicion for malignancy. The arrow indicates the ovarian mass.

**Figure 2:**
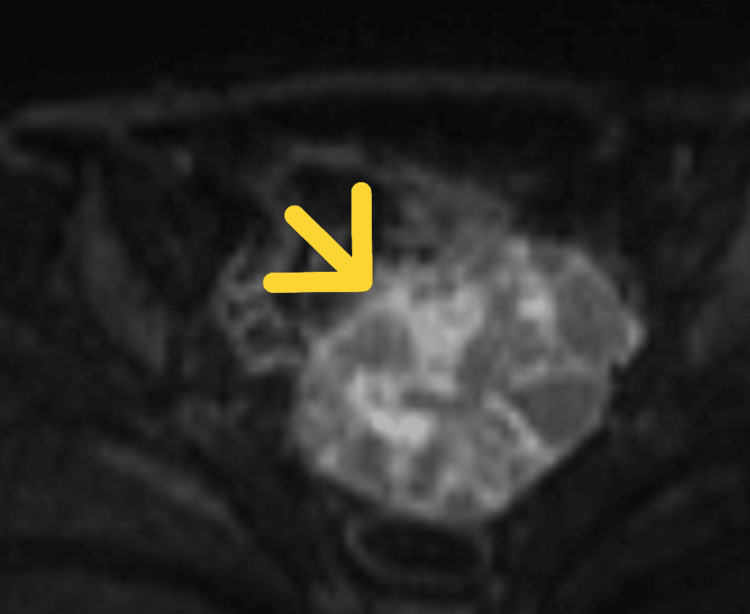
Diffusion-weighted MRI showing the ovarian mass Diffusion-weighted MRI showing the multilocular cystic lesion with focal areas of diffusion restriction, suggestive of high cellularity and supporting the suspicion of ovarian malignancy. The arrow indicates the ovarian mass.

She presented to the gynecology department with a two-month history of progressive abdominal distension. Pelvic ultrasonography revealed a rapidly enlarging left ovarian mass, and pelvic magnetic resonance imaging demonstrated a 106 × 68 × 59 mm multilocular cystic lesion suspicious for malignancy (Figures 1, 2).

Based on these findings, left salpingo-oophorectomy was performed via laparotomy. Intraoperatively, the peritoneal cavity was grossly clean, with no ascites or diffuse peritoneal dissemination. A 5-mm peritoneal nodule suspicious for dissemination and focal induration of the right adnexa were identified; therefore, partial right oophorectomy and resection of the peritoneal nodule were additionally performed. The jejunal tumor was not recognized during this procedure because the operation was primarily focused on the pelvis, and there were no obvious serosal abnormalities or palpable small-bowel mass at that time.

**Figure 3:**
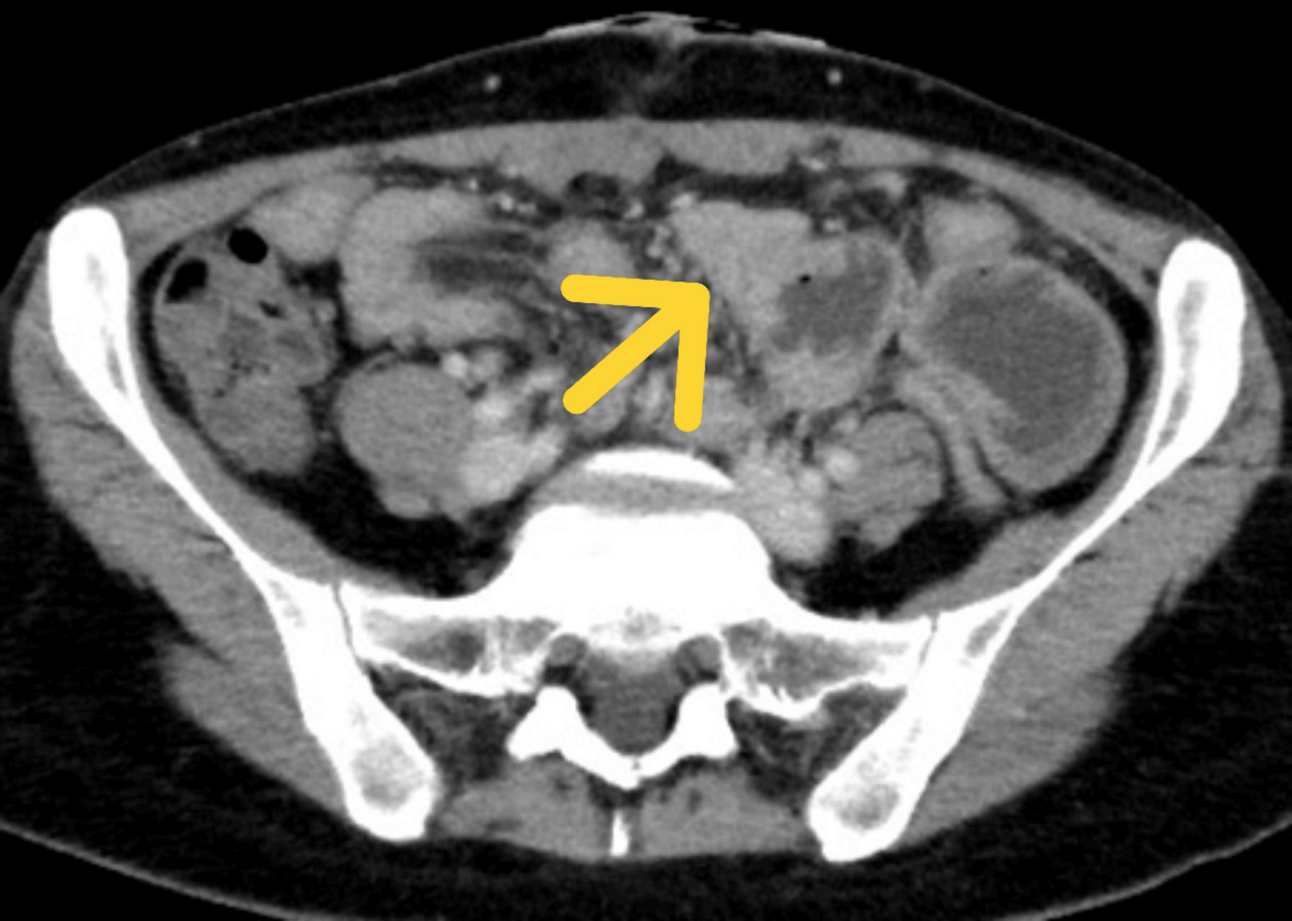
Axial contrast-enhanced CT revealing marked circumferential wall thickening of the upper jejunum The arrow indicates the upper jejunal wall thickening.

**Figure 4:**
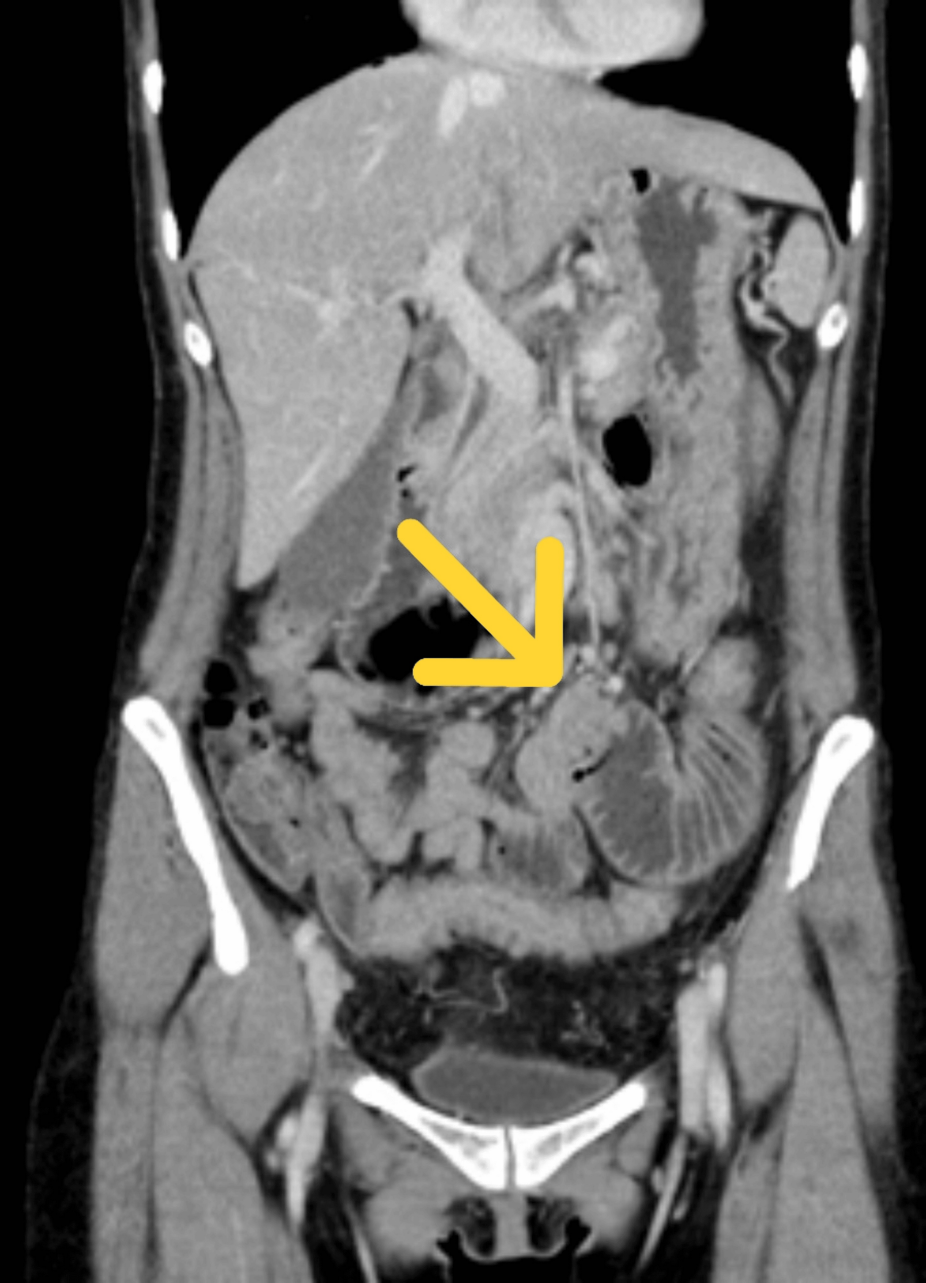
Wall thickening of the upper jejunum Coronal CT reconstruction demonstrating the extent of upper jejunal wall thickening and proximal bowel dilation. The arrow indicates wall thickening of the upper jejunal wall.

On postoperative day 4, the patient developed frequent postprandial vomiting. Contrast-enhanced abdominal CT demonstrated marked circumferential wall thickening of the upper jejunum with proximal bowel dilation (Figures 3, 4).

**Figure 5:**
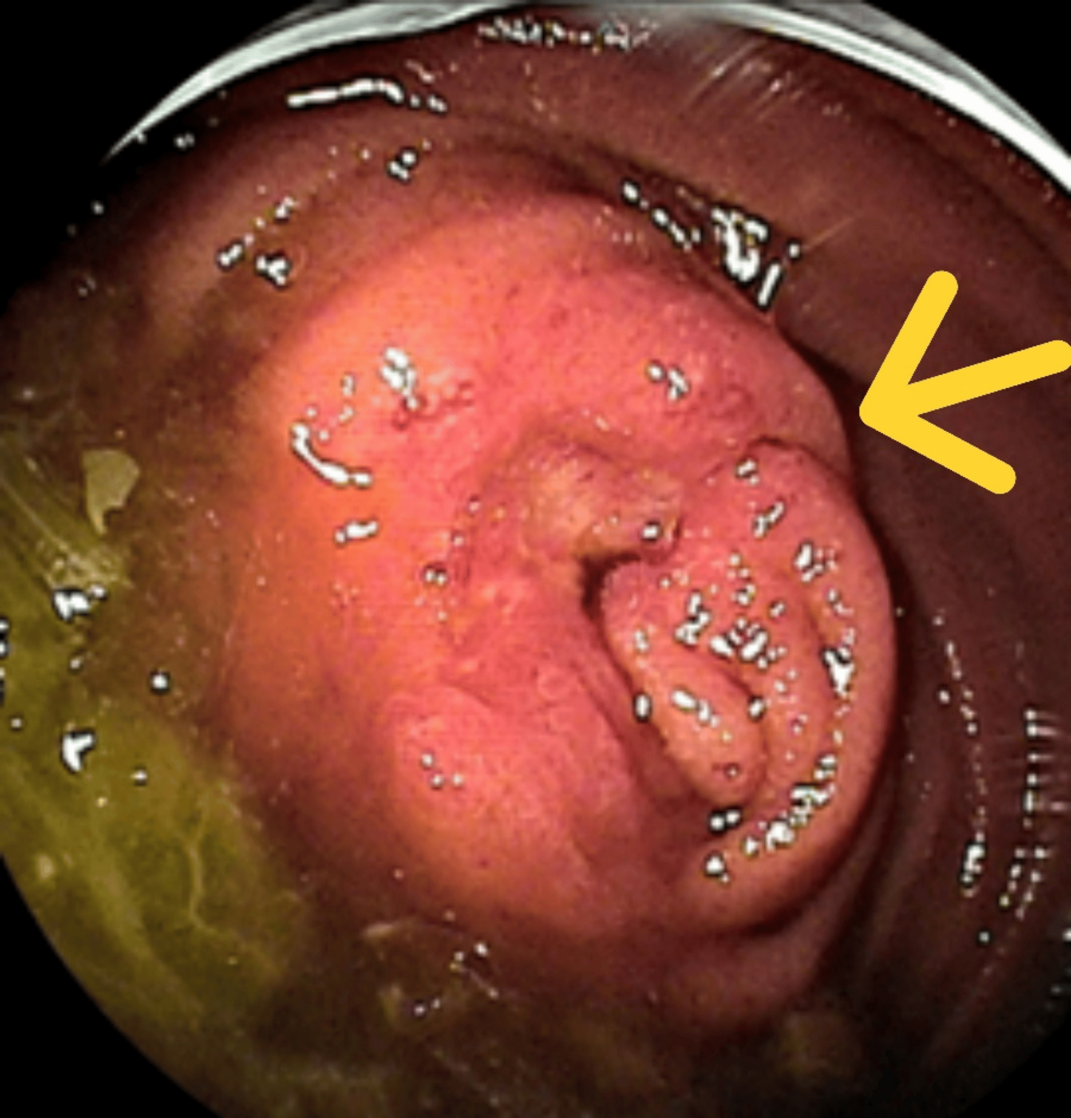
Circumferential ulcerative lesion Small-bowel endoscopy revealed a circumferential ulcerative lesion with impaired passage of the endoscope. The arrow indicates the circumferential ulcerative lesion.

Small-bowel endoscopy revealed a circumferential ulcerative lesion approximately 35 cm distal to the ligament of Treitz, with impaired passage of the endoscope (Figure 5).

Biopsy specimens from the jejunal lesion showed adenocarcinoma. Immunohistochemical staining demonstrated CK7 positivity, CK20 positivity, and CDX2 positivity, with negativity for PAX8, ER, and PgR, supporting a gastrointestinal origin. The ovarian tumor showed a similar immunophenotype, consistent with ovarian metastasis from a primary SBA.

**Figure 6:**
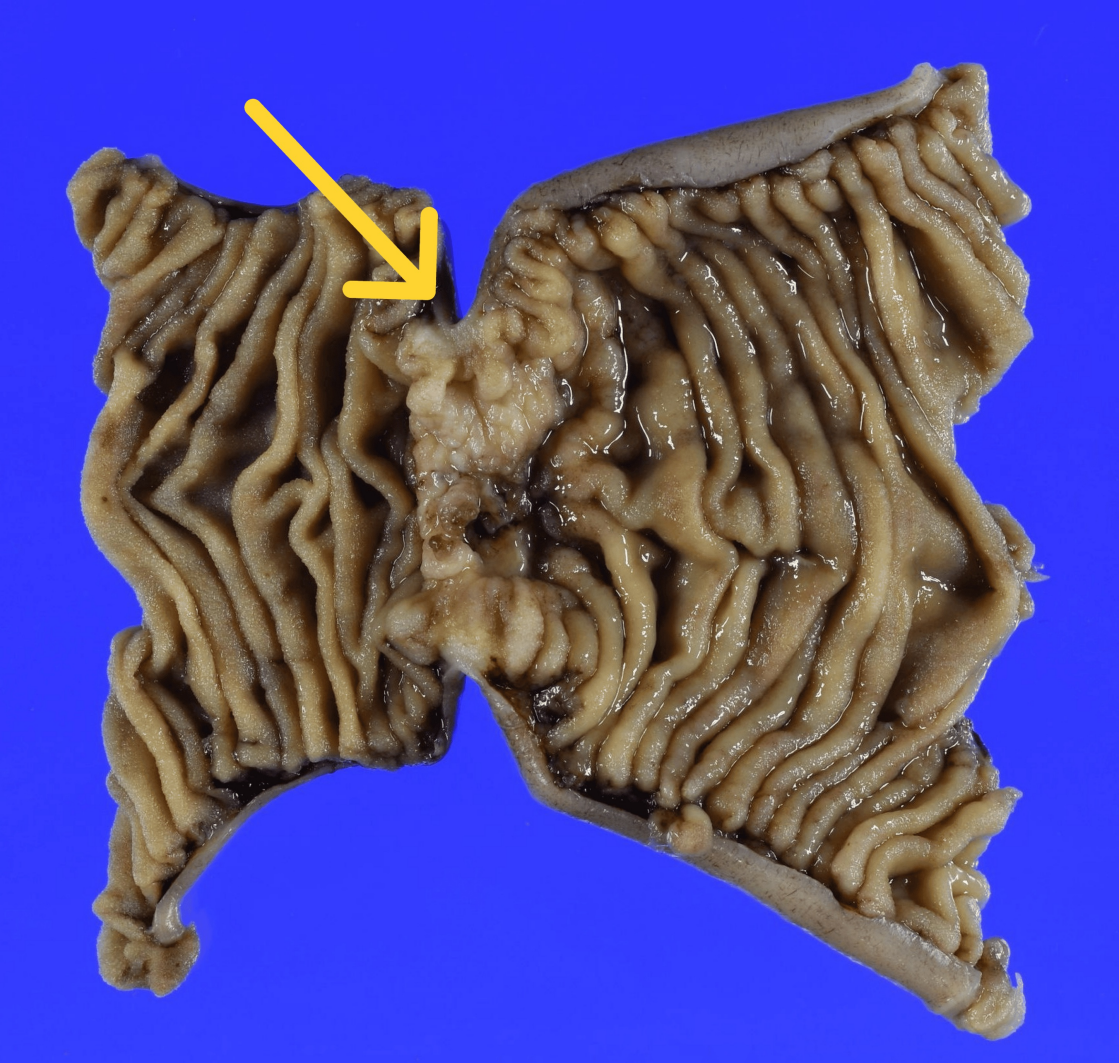
Resected jejunal specimen Resected jejunal specimen showing circumferential stenosis. The arrow indicates circumferential stenosis.

Regarding pre-detection imaging, earlier contrast-enhanced CT had not been performed during routine follow-up because contrast administration was avoided due to her asthma history. The patient subsequently underwent laparoscopic partial jejunal resection (Figure 6).

Regional lymphadenectomy was not performed because the tumor was located in the upper jejunum, and there were no preoperative findings suspicious for nodal metastasis. Additionally, extensive mesenteric dissection was avoided to reduce the risk of ischemia. Gross examination of the resected specimen showed circumferential stenosis (Figure 6). Histopathological evaluation of the surgical specimen confirmed moderately differentiated adenocarcinoma invading through the muscularis propria into the subserosa (pT3). The final diagnosis was primary jejunal adenocarcinoma with ovarian metastasis, classified as pT3 NX M1 (ovary), corresponding to stage IV disease. No malignant cells were detected in the resected peritoneal nodule.

Her postoperative course was uneventful, and she was discharged on postoperative day 8. After discharge, postoperative systemic chemotherapy with capecitabine and oxaliplatin (CAPOX) plus bevacizumab was initiated following colorectal cancer treatment protocols. At the most recent follow-up (18 months after jejunal resection), the patient was alive with disease and continues to receive systemic chemotherapy and regular surveillance.

## Discussion

Primary SBA is a rare malignancy, accounting for less than 1% of gastrointestinal cancers [1]. As its early symptoms are often nonspecific, diagnosis is frequently delayed, and many patients present with advanced disease. Dabaja et al. reported that approximately 50% of patients are diagnosed at Stage III or IV [8]. Common presenting symptoms include abdominal pain, gastrointestinal bleeding, and bowel obstruction [9]. In the present case, the near-complete jejunal obstruction was consistent with an advanced presentation.

Although the duodenum is the most common site of SBA, the jejunum is the second most frequent location in both international and Japanese epidemiologic data [2,9]. Ovarian metastasis from SBA is uncommon but recognized, and several similar cases have been reported in the literature, including clinicopathological analyses and recent case reports [5,8,10]. Compared with previously reported cases, the present case was particularly challenging because the ovarian lesions initially obscured the small-bowel origin. In addition, although the immunohistochemical findings strongly suggested a lower gastrointestinal origin, immunohistochemistry alone cannot completely exclude a gastric or colorectal primary. Therefore, both upper and lower gastrointestinal endoscopies were performed, and no lesions suspicious for another primary site were identified. PET-CT was not performed in this case.

The apparently rapid development of the jejunal lesion despite regular six-month follow-up may be explained by the inherent difficulty of detecting small-bowel tumors during routine surveillance. The small intestine is not easily evaluated by standard endoscopic examinations, and early disease may remain clinically silent. Retrospective review of the previous CT images did not reveal an obvious jejunal lesion. In addition, because the lesion was located in the upper jejunum, identification on preoperative imaging may have been difficult in the absence of a clear mass effect or marked bowel wall abnormality.

Delayed recognition may also be related to the difficulty of identifying small-bowel lesions intraoperatively, particularly when surgery is being performed for another presumed primary pathology. This case, therefore, highlights the importance of considering a small bowel primary when the clinical course is atypical or when the origin of metastatic disease remains uncertain.

Ulcerative colitis is a recognized risk factor for gastrointestinal malignancies. Although its association is strongest with colorectal cancer, several studies suggest that the risk of SBA may also be modestly increased in patients with inflammatory bowel disease [11]. However, standardized surveillance strategies specifically for SBA in patients with ulcerative colitis have not been established. In such patients, careful symptom-based follow-up and timely investigation of new gastrointestinal symptoms may be important for earlier detection.

The prognosis of SBA remains poor, particularly in stage IV disease. Akce et al. reported a five-year survival rate of approximately 10% for stage IV SBA [3], and Japanese multicenter analyses have similarly demonstrated unfavorable outcomes [2]. With regard to treatment, the role of surgery for metastatic disease should be interpreted specifically in the context of SBA. In selected patients with limited and potentially resectable metastatic disease, surgical resection may be considered when feasible, in addition to symptom control [2].

In the present case, jejunal resection was performed as a subsequent operation for postoperative bowel obstruction rather than as part of a planned two-stage strategy. In addition, although lymph node dissection has been described in some reports [12], we did not perform additional lymphadenectomy because wider mesenteric dissection for an upper jejunal tumor raised concern for impaired bowel perfusion and ischemia. Therefore, safe resection for obstruction control was prioritized, and the nodal status was recorded as NX. Postoperatively, systemic chemotherapy based on colorectal cancer treatment protocols, such as 5-fluorouracil (5-FU), leucovorin, and oxaliplatin (FOLFOX) and CAPOX, was administered.

## Conclusions

We report a rare case of primary upper jejunal adenocarcinoma initially diagnosed following resection of an ovarian metastasis and later complicated by high-grade intestinal obstruction. A combination of imaging, endoscopy, and immunohistochemical analysis was essential to distinguish primary SBA from metastatic small-bowel tumors. Timely surgical and oncologic management was crucial for symptom control and overall treatment. This case highlights the diagnostic importance of immunohistochemistry in determining the primary site in patients presenting with Krukenberg tumors of uncertain origin and underscores the need for multidisciplinary management in such rare presentations.
